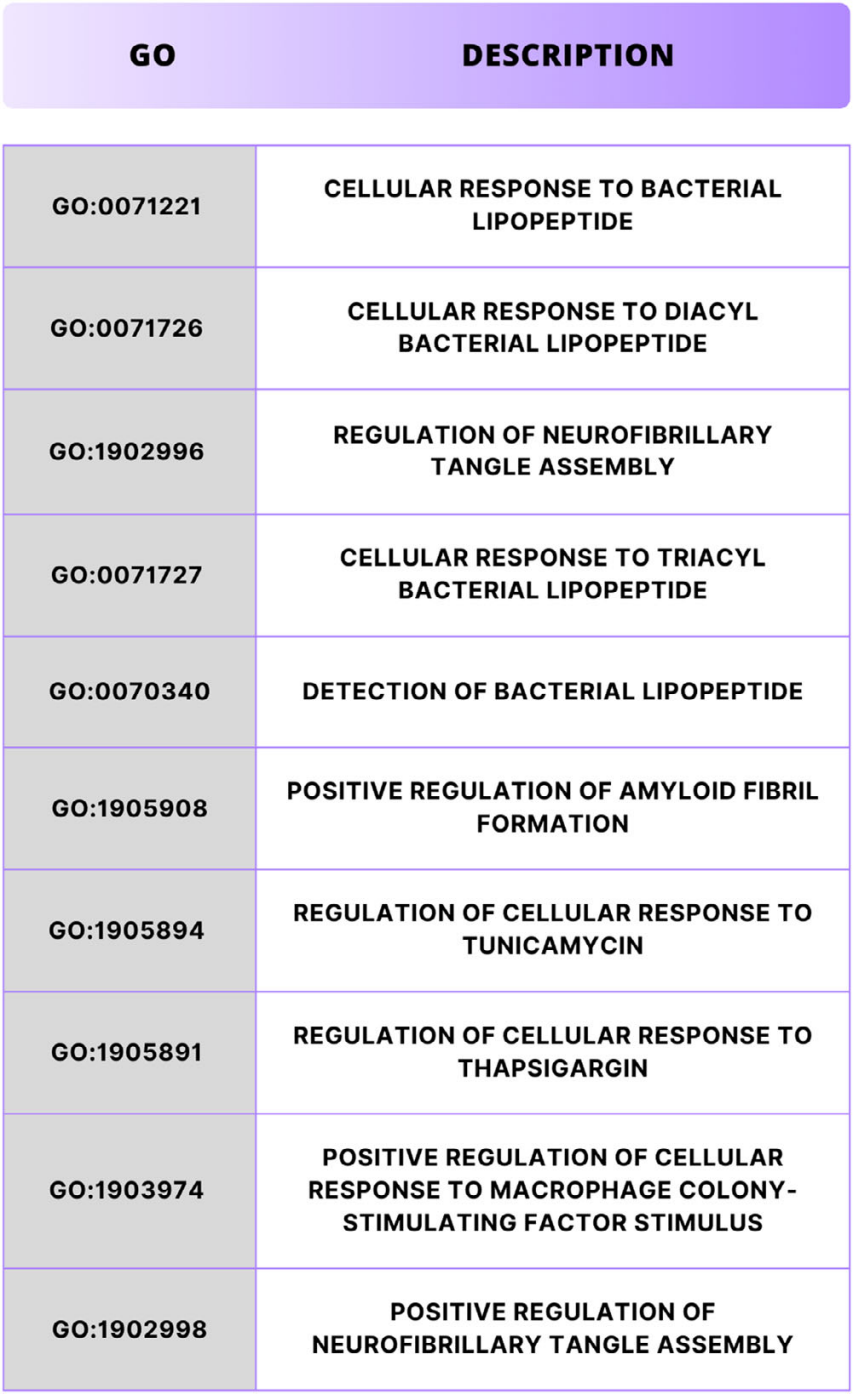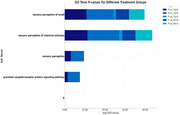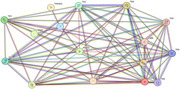# Inflammatory modulation and cellular stress response to lithium treatment in the hippocampus of Alzheimer's disease

**DOI:** 10.1002/alz70855_100586

**Published:** 2025-12-23

**Authors:** Carlos Wagner Leal Cordeiro Júnior, Luara Bela Rocha Gomes, CaíquePortugal de OliveiraCouto, Orestes Vicente Forlenza, Vanessa V.J. De‐Paula

**Affiliations:** ^1^ University of Sao Paulo (USP), São Paulo, São Paulo, Brazil; ^2^ Laboratory of Neuroscience (LIM‐27), Faculty of Medicine, University of São Paulo, Brazil, Brazil, São Paulo, São Paulo, Brazil; ^3^ Faculty UNIRB Teresina, Teresina, Piauí, Brazil; ^4^ Universidade de São Paulo (USP), São Paulo, Brazil; ^5^ University of São Paulo Medical School, São Paulo, São Paulo, Brazil; ^6^ Laboratory of Neurosciences (LIM27), Departamento e Instituto de Psiquiatria, Hospital das Clínicas, Faculdade de Medicina da Universidade de São Paulo, São Paulo, São Paulo, Brazil; ^7^ Laboratory of Neuroscience (LIM‐27), Department and Institute of Psychiatry, Faculty of Medicine, University of São Paulo, Brazil, São Paulo, Brazil; ^8^ USP, São Paulo, Brazil

## Abstract

**Background:**

Alzheimer's Disease (AD) involves chronic neuroinflammation, exacerbated by the activation of Toll‐like receptors (TLRs), such as TLR4, which amplify neurodegeneration through inflammatory cascades. This study evaluated the impact of chronic lithium treatment on the modulation of these inflammatory pathways in transgenic mice (3xTg‐AD).

**Method:**

3xTg‐AD and Wild‐Type (WT) mice were treated with lithium (1 mM and 2 mM) for 8 months. Hippocampi were isolated and analyzed using proteomics via LC‐MS/MS, with protein identification performed using MaxQuant. Cytoscape, STRING, and BINGO were applied for functional, ontological, and protein interaction network analysis. Significant differences were identified using two‐way ANOVA with Benjamini‐Hochberg correction (*p* < 0.05).

**Result:**

The analysis identified 2,807 proteins, of which 932 were exclusive to 3xTg‐AD mice treated with lithium. Among these, proteins associated with NF‐κB and MAPK pathways and TLR4 and IL‐1β—critical components of neurodegenerative inflammation—stood out. TLR4 expression was significantly reduced in the group treated with 2 mM lithium (*p* = 0.003), as was IL‐1β (*p* = 0.005). Protein interaction networks indicated the central role of TLR4 and MAPK1 in clusters related to inflammatory modulation and cellular stress response. Functional ontology analysis highlighted enrichment in terms such as “regulation of immune response” (*p* = 3.63E‐12) and “stress response” (*p* = 2.22E‐4). Visualizations generated in Cytoscape revealed 35 proteins as central hubs, demonstrating a strong relationship between TLR4 and tau and beta‐amyloid proteins, indicating that lithium directly interfered with inflammatory signaling associated with AD.

**Conclusion:**

Chronic lithium treatment effectively modulated neuroinflammation in Alzheimer's transgenic mice by reducing pro‐inflammatory markers and reorganizing molecular networks associated with TLRs, particularly TLR4. These findings reinforce lithium's potential as a therapeutic modulator, though further investigations are needed for translation to human clinical studies.